# Quality control, best practices and variability of IVF results

**DOI:** 10.5935/1518-0557.20200021

**Published:** 2020

**Authors:** David Meldrum

**Affiliations:** 1 Reproductive Partners San Diego, San Diego, California, USA; 2 Division of Reproductive Endocrinology and Infertility, University of California, San Diego, California, USA

As an attendee at various national and international conferences during the formative years of IVF in the early 1980’s, I distinctly remember how difficult it was to hear a myriad of laboratory and clinical techniques being described and to have any possibility of discerning which were best practices for IVF success. I concluded that the only way to be successful was to copy every aspect of the process from a successful program. I therefore took a sabbatical in Melbourne Australia with Alan Trounson and his clinical team at Monash, while also observing another successful program across town at The Royal Women’s Hospital with Alex Lopata. That experience convinced me of the critical role of a top quality IVF laboratory and allowed me to establish a highly successful IVF program on my return to UCLA (Meldrum *et al*., 1987).

A few years later as President of the Society for Assisted Reproductive Technology (SART), I helped to set up a program with the College of American Pathologists (CAP) for accreditation of embryo laboratories in the U.S. and served as the first commissioner. However, the CAP model focused on quality control aimed at assuring accurate and reproducible test results, whereas the successful outcome of an IVF laboratory is a healthy baby. In the absence of any widely accepted best practices I realized that the most we could do was to develop an accreditation program emphasizing quality control. As just one example, a quality laboratory must have written procedures and an accreditation visit must verify that those procedures are being followed. 

Recognizing the limitations of existing clinical conferences and the laboratory accreditation process, I realized that the best way to improve IVF results was to set up a postgraduate course with lectures on every aspect of IVF-ET by recognized authorities having good outcomes. Due to the world pandemic, July 18-21, 2021 will be be our 33^rd^ year directing this University of California course, now on Coronado Island in San Diego in conjunction with the University of California San Diego. It currently attracts over 400 attendees, the majority of whom are from other countries (www.davidmeldrummd.com - click on “Coronado Island”).

The practice committee of ASRM has assumed the ultimate task in this process by beginning to publish best practices, as recently illustrated with their publication on embryo transfer (Penzias *et al*., 2017). That process relies on a detailed review of the literature and a consensus forged by a group of experienced clinicians. Use of an embryo transfer simulator is a new important effort to optimize training in this most critical aspect of IVF. However, because chosen research papers may not fully reflect best practices, it remains my strong belief that copying all aspects of techniques used by a highly successful practitioner ads a further level of assurance. Dr. William Schoolcraft, who has some of the best IVF results in the U.S. and introduced routine ultrasound guided transfer into his program several years before others (Schoolcraft *et al.*, 2001), has given the Coronado lecture on embryo transfer for many years.

Still, with all these efforts we have a long way to go to make results in individual IVF clinics more uniform. For my introduction to the first “fertile battle” in *Fertility and Sterility* I reviewed individual outcomes published by SART and cautioned that we could not ignore the “elephant in the room” (Meldrum, 2018). Clinics’ implantation rates (IRs) varied from 11 to 83%, starkly showing that there is a wide variation of IVF program quality (a program’s IR is arguably a good objective index of overall best practices, albeit influenced by the proportion of cycles having Preimplantation Genetic Testing for Aneuploidy: PGT-A).

SART has also begun a service to send experienced individuals to provide onsite help to improve laboratory and clinical procedures for a member program requesting help because of an unsatisfactory success rate. While costly, that may be the best way to jump-start a program with more serious problems. Early on in IVF I visited 15 to 20 programs experiencing unsatisfactory outcomes, mostly in academic settings. A thorough review always turned up significant issues that were simple to correct. I was recently reminded by a Harvard professor that when I had done a site visit there early in the evolution of IVF, I had told the fellows (of which he was one) that there are about a hundred things one has to get right to have a highly successful IVF program. Onsite visits are not something I have chosen to spend my time on since then, but I found it very satisfying.

The most important factor is the insight of individual program directors and their fellow physicians to accept their limitations and to be sufficiently motivated to spend the time to learn. Time out of the office to attend a course such as ours and time to read the journals can easily take second place to other demands of busy lives. They also might convince themselves that they have more difficult patients or use other excuses to explain away their lower results. Over the years I have had many experienced physicians attend our course looking for any improvements they can glean. It has been particularly satisfying to have had physicians from Melbourne, where I was first launched into the field. IVF practitioners should not be too proud to ask for help or to be seen at a course learning. IVF is a very “tough nut to crack”.

Patient education is the most potent force to stimulate programs with suboptimal results to improve. SART membership gives some reassurance. It is not awarded to a programs without agreeing to have their laboratories certified and to publish their results subject to an on-site audit. Hopefully all other international societies will require the same. Couples seeking IVF care must carefully assess the qualifications and results of physicians and programs under consideration. The field of IVF has been more strictly regulated than most others, but consumers must do their homework, which information technology makes easier than ever.
